# Fluorimetric Immunoassay for Multianalysis of Aflatoxins

**DOI:** 10.1155/2013/584964

**Published:** 2013-08-13

**Authors:** Lizy Kanungo, Sunil Bhand

**Affiliations:** Biosensor Lab., Department of Chemistry, BITS, Pilani-K. K. Birla Goa Campus, Goa 403726, India

## Abstract

A sensitive fluorimetric ELISA was developed for the analysis of aflatoxins. The assay was performed in a 384 microwell plate, wherein high specificity monoclonal antibody against AFM1 (mAb-AFM1) was used as capture antibody and FITC conjugated secondary antibody was used for detection and quantification of the analyte. The linear range of the immunoassay was found to be 6.25–50 pg/mL. AFM1 as low as 1 pg/mL was detected by this method with assay volume 40 **μ**L. The multi-analysis of different aflatoxins was also investigated in the microwell plate, based on the cross-reactivity (CR) approach. Real milk samples were tested along with certified reference material by standard addition method and recovery analysis was done. The mAb-AFM1 showed 23.2% CR with AFB1, 50% CR with respect to AFM2, and least CR towards AFG1 (<1%). Furthermore, mixture analysis of AFM2 and AFB1 was carried out at specific concentrations of AFM1. The advantages of this developed immunoassay are high sensitivity, high throughput, multianalyte detection, versatility, and ease of handling.

## 1. Introduction

Aflatoxins are highly toxic, mutagenic, carcinogenic, and teratogenic compounds contaminating a wide range of food commodities [1, 2]. The major naturally occurring aflatoxins, namely, AFB1, AFB2, AFG1, and AFG2, constitute a class of structurally related toxic fungal metabolites. They are extremely potent carcinogens and can have significant economic impacts, making them important targets for detection and quantification [3]. Commodities frequently contaminated by aflatoxins include cereals, nuts, dried fruits, spices, and pulses [4, 5]. When animals consume AFB1 contaminated foodstuffs, the toxin is metabolized in the liver and excreted as AFM1 via milk and urination [1]. Humans are exposed to the deleterious effects of aflatoxins either directly by eating contaminated grains or indirectly via animal products [6]. Evidence of hazardous human exposure to aflatoxins through various foods including dairy products has been shown by several investigators [1–3, 7]. 

Many analytical methods have been developed for estimation of aflatoxins in agricultural commodities. Among them optical and electrochemical transducers are most widely used for detection of aflatoxins. The optical detection method is regarded as one of the sensitive techniques for aflatoxin analysis. The optical signal is not influenced by electrical, magnetic, or ionic fields.  Table 1 gives an account of some of the reported biosensors for analysis of AFM1 and AFB1.

**Figure 1:**
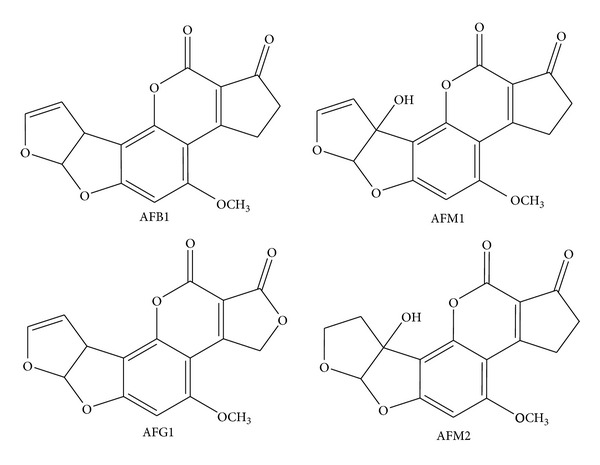
Chemical structures of different aflatoxins.

It is evident that the need for a highly selective multianalyte detection system is acute. Simultaneous analysis of aflatoxins is essential to minimize the consumption of contaminated food and feed and import surveillance programs and controlling quality of products [5, 8, 9]. There is a need for development of single platform for simultaneous analysis of these toxins. Few attempts have been made towards development of immunosensors capable of both discriminative detection and class-selective multianalyte analysis. In one such approach, multianalysis of aflatoxins was investigated by development of an immunoaffinity column. This was done by covalently coupling monoclonal antibody 1C11 against aflatoxins to amino-silica gel microparticles and then packing these into a cartridge. This immunoaffinity column was subsequently used for selective extraction of aflatoxins in agri-products [10]. In a recent article, novel peptide mimics of aflatoxin (mimotopes) have been used for analysis of aflatoxins [11]. In this work, 5 such mimotopes were obtained and used in an indirect competitive ELISA for analyzing total aflatoxin concentration in peanut and feedstuffs. In another interesting article, multianalysis of different triazine herbicides using CR of antibodies coupled to pattern recognition has been reported [12]. A similar approach could be implemented for multianalysis of different aflatoxins which are structurally similar (Figure 1). 

**Figure 2:**
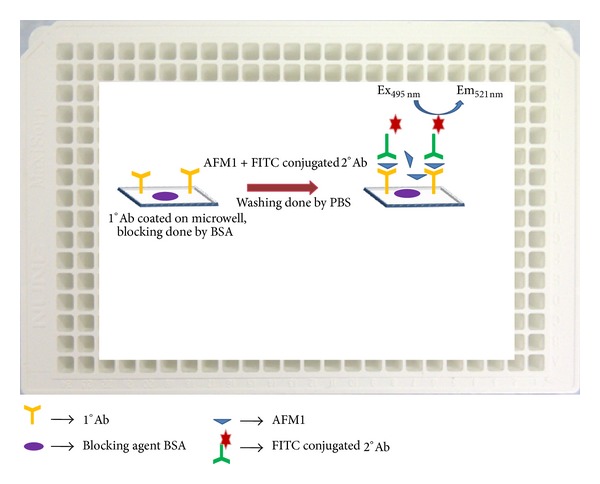
Scheme of fluorimetric ELISA designed for analysis of AFM1.

In this work, a simple ELISA was developed using fluorimetric technique. We performed fluorimetric analysis of AFM1 using FITC conjugated secondary (2°) antibody in 384 microwell plate as shown in Figure 2. Multianalysis of different aflatoxins was carried out by CR approach. The microwell plate was coated with anti-AFM1 primary (1°) monoclonal antibody (mAb) which reacted with AFM1 and partly recognized structurally similar aflatoxins such as AFM2, AFB1, and AFG1. Subsequently we analyzed 2 real milk samples using standard addition method, and recovery rate was studied. The CR was studied and mixture analysis was carried out. The occurrence of cocontaminants was detected by this mixture analysis.

## 2. Experimental 

### 2.1. Chemicals and Instrumentation

AFM1, bovine serum albumin (BSA), Tween 20, and certified reference material (CRM) ERM-BD282 (AFM1 in whole milk powder, <0.02 *μ*g/kg) were obtained from Sigma-Aldrich (USA). AFB1, AFG1 were purchased from Acros Organics, USA. AFM2 was purchased from Fermentek, Israel. Acetonitrile (ACN) HPLC grade and sodium chloride (NaCl) were purchased from Merck (Germany). Sodium hypochlorite (4%) solution was purchased from Fisher Scientific (India). Rat monoclonal [1C6] primary antibody (1°Ab) of AFM1 and FITC conjugated 2°Ab were purchased from Abcam (UK). Milk samples were centrifuged by MiniSpin Plus centrifuge purchased from Eppendorf (Germany) and shaking of the samples were done by Spinix shaker, purchased from Tarsons (India). 0.22 micron filter papers (25 mm diameter) obtained from Millipore (USA) were used for filtration of milk samples. White 384 well polystyrene microtiter plates were purchased from Nunc (Denmark). For fluorimetric measurement, VictorX^4^ 2030 OptiPlate reader from Perkin Elmer (USA) was used. A glove box, Cole Parmer (USA), was used for the handling of all aflatoxin standard solutions. Water produced in a Milli-Q system (Millipore, Bedford, MA, USA) was used to prepare all the solutions. pH meter from SevenMulti Mettler Toledo, Switzerland, was used to prepare buffers. For sample handling, micropipettes (Eppendorf, Germany) were used. Milk samples were purchased from the local markets of Goa, India. The data processing was done by Origin 6.1 (Microcal, USA). 

### 2.2. Preparation of Buffers

Carbonate buffer (CB; 0.05 M, pH 9.6), phosphate buffered saline (PBS; 0.01 M, pH 7.4), PBST (PBS with Tween 20), and blocking solution (BSA in PBS) were made by the same protocol as described in our earlier paper [27]. All buffer solutions were stored at 4°C when not in use. 

### 2.3. Preparation of Aflatoxin Standard Solutions

All the aflatoxin solutions were prepared in a glove box in a maintained inert (N_2_) atmosphere. AFM1 stock solution was prepared by dissolving the AFM1 powder in 5% ACN (v/v) in PBS at a concentration of 5 *μ*g/2 mL and stored at 2–8°C. Working standard solutions in the range of 1–500 pg/mL were prepared by diluting the stock with 5% ACN. AFM2 standard solutions were also made in the similar way. AFB1 stock solution 1000 *μ*g/mL was prepared by dissolving the crystalline AFB1 in 5% ACN (v/v) in PBS and stored at 4°C. Working AFB1 standard solutions were prepared in the following concentrations: 1, 6.25, 12.5, 25, 50, 100, 250, and 500 pg/mL by diluting the stock with 5% ACN. AFG1 standard solutions were also made in the similar manner. (Safety note: aflatoxins are highly carcinogenic and should be handled with extreme care. Aflatoxin contaminated labware should be decontaminated with an aqueous solution of sodium hypochlorite (4%).)

### 2.4. Preparation of Aflatoxin Antibody Solutions

The stock solution of rat monoclonal [1C6] 1°Ab, 100 *μ*g (1 mg/mL) was diluted with 50 *μ*L of pyrogen free deionized water. It was divided into 2 fractions. The first fraction containing 40 *μ*L was stored at −20°C. From the second fraction, the working antibody dilutions were prepared by serial dilution in double distilled water as 1 : 1000, 1 : 2000, and so forth and then added with equal volume of CB (1 : 1). From the FITC labeled 2°Ab stock 1 mg (2 mg/mL) solution, 100 *μ*L was diluted with 500 *μ*L of deionized water. It was divided into 2 fractions. The first fraction containing 400 *μ*L was stored in −20°C. From the second fraction, working 2°Ab solution was prepared prior to the experiment by serial dilution in PBS as 1 : 1000, 1 : 2000, and so forth. 

### 2.5. Milk Sample Pretreatment

CRM-BD282 milk powder comprising zero level AFM1 was reconstituted in the following manner. One g of CRM-BD zero level milk powder was dissolved in 10 mL of warm PBS. Then it was centrifuged at 6000 rpm for 10 min. The upper fatty layer was completely removed, and middle fat free portion was taken out. The decanted aqueous layer was filtered through a syringe filter using 0.22 micron filter paper. This was further diluted with PBS in 1 : 1 manner and used for analysis. Packaged milk samples containing 3.5% fat were collected from the markets of Goa. These packaged milk samples were centrifuged, filtered, and treated in the same manner. 

### 2.6. Fluorimetric Immunoassay Procedure

ELISA was performed in a 384 microwell plate. 1°Ab was diluted to 1 : 16000 in CB and coated as 40 *μ*L/well. The plate was covered with parafilm and aluminium foil, kept at 4°C for overnight, and washed 3 times by rinsing the wells with 40 *μ*L PBS. The remaining protein binding sites in the coated wells were blocked by adding 40 *μ*L of blocking solution for about 1 h at room temperature. The wells were washed twice with 40 *μ*L PBST. Following this step, AFM1 standard solution in the range 1–500 pg/mL was mixed up (at equal volume) separately with optimized 2°Ab (diluted to 1 : 64000 in PBS) solution. This antigen-antibody mixture solution was then added as 40 *μ*L/well. The plate was incubated for about 2 h at room temperature. The excess label was removed by washing with PBS. The microplate was then analyzed by VictorX^4^ 2030 optiplate reader in fluorescence mode. 

## 3. Results and Discussion

### 3.1. Optimization of Antibody Dilutions

**Figure 3:**
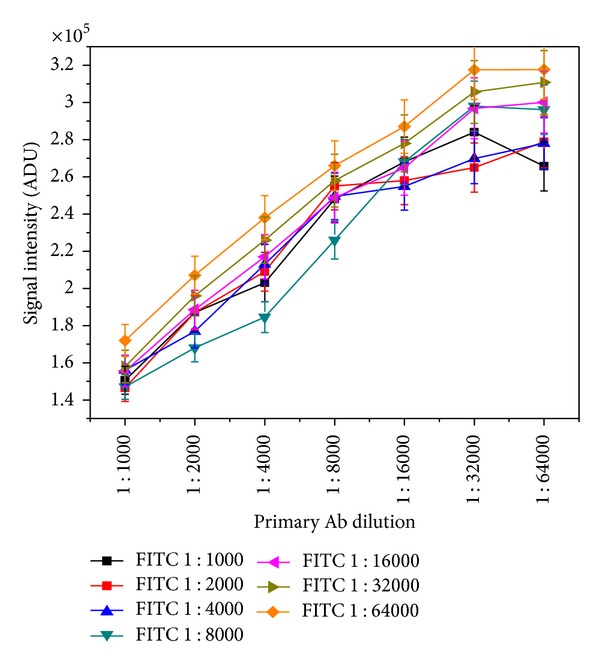
Optimization curve of FITC labeled 2°Ab against 1°Ab.

The FITC labeled 2°Ab solutions were made by serial dilution from 1 : 1000 up to 1 : 128000.  Figure 3 shows the optimization result obtained for FITC labeled 2°Ab with 1°Ab in fluorimetric assay. It was observed that FITC labeled antibody at 1 : 64000 dilution showed best signal (3.2 × 10^5^) count when assayed with 1°Ab. The excitation time was 0.1 s.

### 3.2. Optimization of Incubation Time

**Figure 4:**
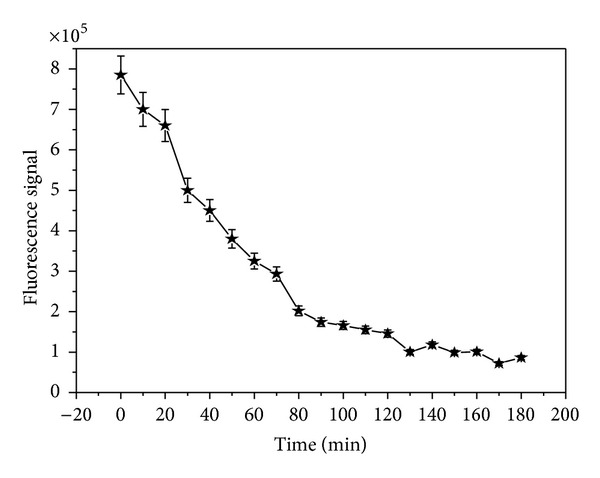
Optimization of incubation time for FITC conjugated 2°Ab. 1°Ab dilution, 1 : 16000; FITC conjugated 2°Ab dilution, 1 : 64000; excitation time, 0.1 s.

In order to achieve the optimal binding of analyte, the incubation time varied from 10 min to 180 min.  Figure 4 shows the optimization result of incubation time of AFM1 and FITC labeled 2°Ab complex with capture 1°mAb. It is apparent from the graph that around 100–120 min, the stable signal was obtained. So, the incubation time was chosen at 120 min in further experimental analysis.

### 3.3. Calibration of AFM1

**Figure 5:**
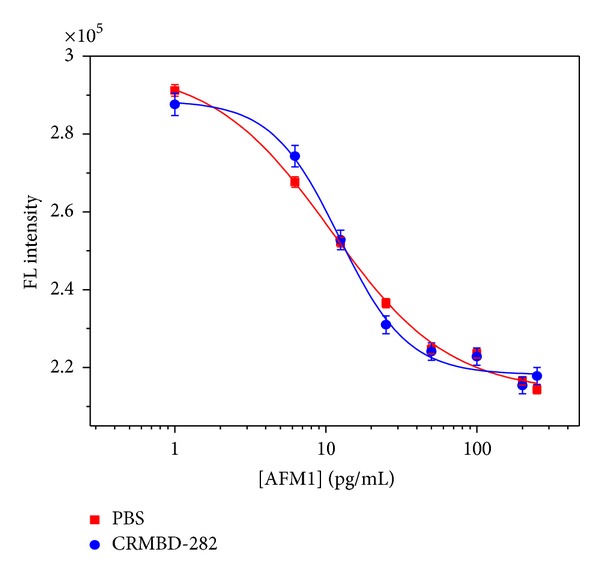
Calibration curve of AFM1 obtained using fluorimetric assay in buffer and CRM-BD282. 1°mAb dilution, 1 : 16000; FITC conjugated 2°Ab dilution, 1 : 64000; incubation time, 2 h; excitation time, 0.1 s.

Different concentrations of AFM1 were tested both in PBS and in CRM-BD282 zero level AFM1 milk powder. Calibration was studied in the range 1–250 pg/mL by fluorimetric analysis.  Figure 5 shows graph of fluorimetric signals obtained for varying concentrations of AFM1 in PBS and CRM-BD282. 

In both cases, decrease in the signal intensity was observed with the increase of [AFM1]. The fluorimetric assay could quantify the [AFM1] in an ultrasensitive manner with a lower limit of detection (LOD) at 1 pg/mL. The analytical figures of merit of this AFM1 assay by fluorimetric technique are given in Table 2. 

### 3.4. Fluorimetric Immunoassay for Multianalysis of Aflatoxins

It is evident that aflatoxins such as AFM1, AFM2, AFB1, and AFG1 have similar structures (Figure 1). Thus the capture antibody that recognizes its specific analyte may also partly recognize the other structural analogues. Bearing this concept in mind, CR studies were carried out for AFM1, AFB1, AFG1, and AFM2 recognition by the 1°mAb. The anti-AFM1 mAb was used as capture antibody, and FITC conjugated antibodies were used as 2°Ab for recognition of antigen-antibody (Ag-Ab) complex. The multianalysis of different aflatoxins using the CR with mAb of AFM1 was studied keeping the competitor concentration constant in all measurements.

The standard solutions of different aflatoxins such as AFM1, AFM2, AFB1, and AFG1 were prepared ranging from 1 to 500 pg/mL. These concentrations were mixed up with FITC labeled 2°Ab in equal volume (total 40 *μ*L) and added to four different rows of the microwell plate. After 2 h of incubation, washing was done to remove any unbound antigen or antibody. Then the plate was analyzed in the multiplate reader. 

**Figure 6:**
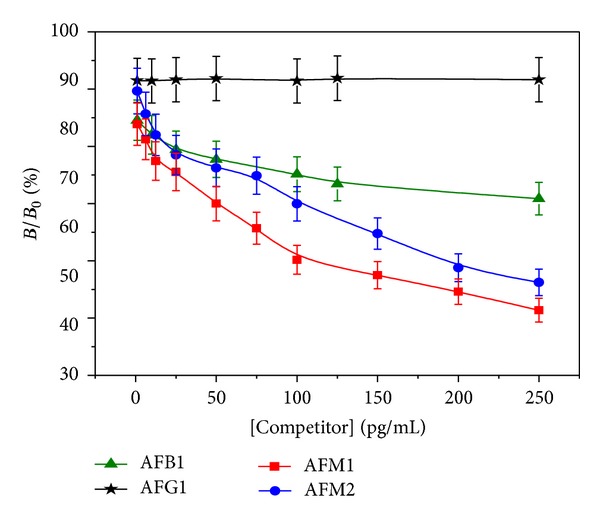
Fluorimetric analysis of aflatoxins at different concentrations carried out using 1 : 16000 1°mAb in CB as capture antibody, FITC labeled 2°Ab used at 1 : 64000 dilution; incubation time was 2 h, excitation time 0.1 s.

Figure 6 shows the different aflatoxins specific recognition towards the mAb of AFM1 where concentration of competitor was plotted against *B*/*B*_0_ (%). The CR study result (Figure 6) of fluorimetric assay shows that FITC labeled anti-AFM1 antibody showed strong intensity when recognized AFM1-Ab complex. It recognized other aflatoxins differentially. With the increase in [AFM1], the signal intensity decreased due to inhibition.

The CR of mAb pair was determined in competition with tracer. For standard analyte (for which CR is 100%), concentrations that result in 50% inhibition (IC_50_) of the signal were obtained from calibrations and were used to compute the CR [12] using the formula

(1) $$\begin{array}{c} \text{CR}\left(\%\right)=\frac{{\text{IC}}_{50}\;\text{value}\;\text{of}\;\text{standard}\;\text{analyte}}{{\text{IC}}_{50}\;\text{value}\;\text{of}\;\text{cross}\;\text{reacting}\;\text{analyte}}\times 100. \end{array}$$

To evaluate the sensitivity of the ELISA, the IC_50_ was obtained from the standard curves. The specificity of the ELISA was evaluated by determining the CR with structurally related aflatoxins. The mAb of AFM1 was highly specific to AFM1 and showed partial recognition towards its structural analogues AFM2 and AFB1. Figure 6 shows different aflatoxins specific recognition towards the mAb of AFM1 where concentrations of competitor were plotted against *B*/*B*_0_ (%), where *B*_0_ is the maximum signal obtained in absence of analyte and *B* is the signal obtained in presence of analyte concentration.  Table 3 summarizes the *B*/*B*_0_% of different aflatoxins. 

The IC_50_ values of AFM1 and AFM2 were obtained, but for AFB1 and AFG1, IC_50_ could not be obtained as its CR with AFM1 mAb was not established. As indicated in Table 3, the mAb of AFM1 showed 23.2% CR with AFB1, approximately 50% with AFM2 and showed negligible (<1%) CR with AFG1. The standard curve of AFM1 was used as reference in the ELISA as indicated in Figure 6. 

### 3.5. Real Sample Analysis

The real sample analysis for two milk samples was carried out using mAb against AFM1 as receptors. The concentrations of AFM1, AFM2, AFB1, and AFG1 were determined in the two milk samples using CR approach coupled with standard addition method. The wells were coated by mAb of AFM1 and incubated. Then washing was done with PBS followed by blocking by BSA. In four different rows, the CRM-BD282 zero level AFM1 milk samples were spiked with 50 pg/mL of AFM1, AFB1, AFG1 and AFM2, respectively. Then milk samples 1 and 2 were added to the wells followed by FITC conjugated Ab. After incubation of about 2 h and washing with PBS, they were analyzed by multiplate reader. All experiments were made in triplicate. The recovery data are given in Table 4. The precision was determined by calculating the relative standard deviation (R.S.D.%) for the replicate measurements. The R.S.D.% and R.E.% were calculated by the following formulae:

(2) R.E.  (relative  error)% =measured  value−true  valuetrue  value×100; n=3,R.S.D.  (relative  standard  deviation)% =standard  deviationmean×100; n=3.

Results, as listed in Table 4, showed an acceptable recovery in the range of 90%~101%, and R.S.D. was from 0% to 8%. It was noted that the CRM milk samples spiked with 50 pg/mL of AFM1 showed 100% recovery. In S1, higher recovery was obtained as compared to S2. In case of AFM2, the recovery was found to be 91%. Here also, S1 showed higher recovery than S2. For AFB1, the recovery was found to be 81%, and when tested for S1 and S2, it showed 100.8% and 93.6%, respectively. Although in S1 analysis for AFB1, >100% recovery was obtained, the concentration (400 pg/mL) was much below the recommended level (2 *μ*g/L). For AFG1 analysis, the recovery was 100.4%. In this case, the recovery for S1 and S2 was obtained as 92.6% and 93.6%, respectively. 

### 3.6. Mixture Analysis

**Figure 7:**
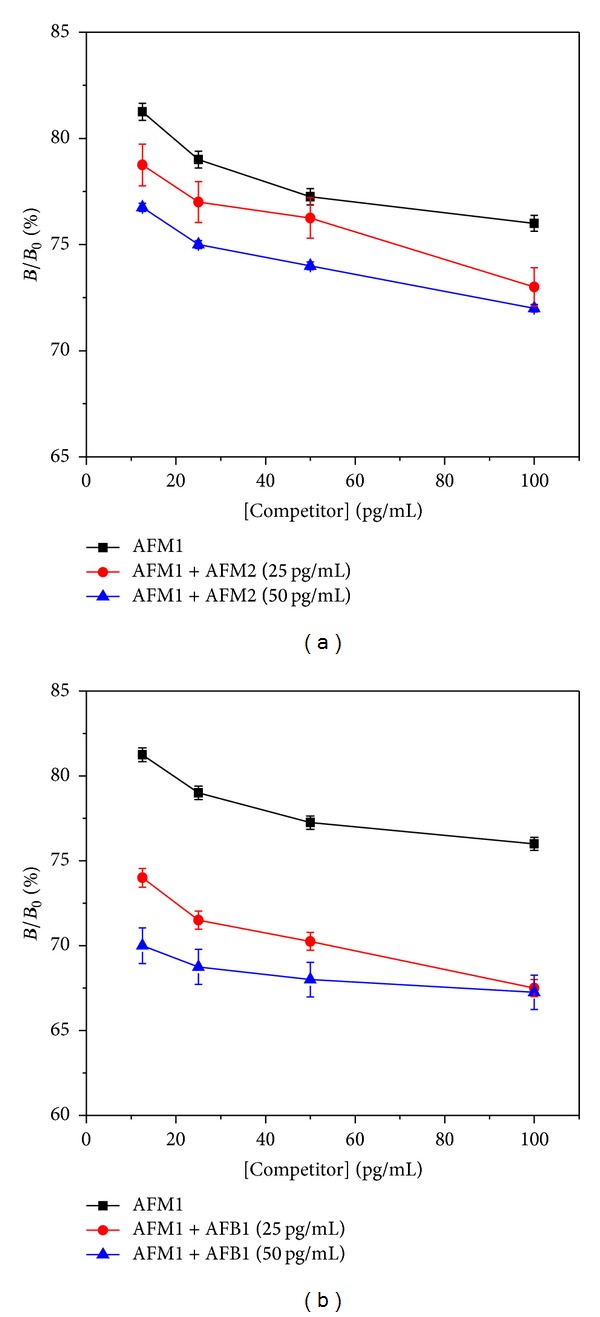
Mixture analysis by fluorimetric immunoassay: (a) AFM1 and AFM2 and (b) AFM1 and AFB1.

The CR studies were further carried out using mixture analysis. The mixture analysis was done by adding 50 and 25 pg/mL of AFM2 and AFB1 (individually) in varying amount of AFM1 in the range 1–100 pg/mL as shown in Figures 7(a) and 7(b).

It was observed that, at 12.5 pg/mL of AFM1, the AFM1 + AFM2 (25 pg/mL) mixture showed 4% and the AFM1 + AFM2 (50 pg/mL) mixture showed 10% lesser signal intensity than that of only AFM1. Similarly at 25 pg/mL of [AFM1], the mixture of both AFM1 + AFM2 (25 and 50 pg/mL) showed 2% and 4% decreases in signal intensity, respectively, as compared to only [AFM1]. At the EU cutoff limit or 50 pg/mL, there was no significant variation observed. Only 1.5% of decrease in signal intensity was obtained for AFM1 + AFM2 (25 pg/mL) mixture. But when analyzed for AFM1 + AFM2 (50 pg/mL) mixture, it showed 4% lesser signal when compared to only AFM1. At higher concentration of AFM1 (100 pg/mL), the AFM1 + AFM2 (25 pg/mL) showed 4% and the AFM1 + AFM2 (50 pg/mL) showed 5% lesser signal intensity when compared to that of only AFM1. This mixture analysis postulates an account of some relation between CR and signal suppression. AFM2 showed almost 50% CR with AFM1, and its presence as cocontaminant in the mixture resulted in small variation of 1.5 to 4% decrease at lower concentration (25 pg/mL), whereas at 50 pg/mL, the signal suppression was observed in the range of 4 to 10% variation.

The mixture analysis was also carried out for AFB1 and AFM1 using anti-AFM1 antibody as shown in Figure 7(b). The standard calibration curve for AFM1 was plotted against mixture of AFM1 and AFB1 (25, 50 pg/mL). In this case, further decline in signal intensities was observed. At 12.5 pg/mL of [AFM1], [AFB1] for 50 and 25 pg/mL showed 7.5% and 11.5% decrease in signal, respectively. For 25 and 50 pg/mL of [AFM1], the AFM1 + AFB1 (25 pg/mL) mixture showed the same decrease in signal of about 8%, but for AFM1 + AFB1 (50 pg/mL) mixture it was 11% and 9.5%, respectively. At higher concentration of AFM1 (100 pg/mL), the AFM1 + AFB1 (25 pg/mL) and AFM1 + AFB1 (50 pg/mL) mixtures showed 9 and 9.5% lesser intensities, respectively. The curve representing the mixture of [AFM1] with 100 pg/mL [AFB1] showed 12 and 10% less signal intensity of 25 and 50 pg/mL, respectively, when compared to only [AFM1]. In all the mixtures of AFB1, the signal suppression was observed in a higher order of 7.5 to 11.5% variation when compared to the mixture analysis of AFM2. This difference has occurred for the reason of difference in CR of AFB1 with AFM1 (23%) which differs from 50% CR of AFM2 with AFM1. This mixture analysis can play significant role in detecting and differentiating the presence of different aflatoxins which exist as cocontaminants found in the food commodities. Based on their CR properties, AFM1, AFM2, and AFB1 can be detected and quantified if found mixed in food samples.

## 4. Conclusions

This work illustrates a simple and sensitive fluorimetric multianalyte microwell plate based immunoassay for aflatoxins. Ultrasensitive analysis of AFM1 was successfully carried out by the developed technique with LOD as low as 1 pg/mL. The presented protocol has been improved by eliminating one step in the immunoassay. ELISA for multianalysis of different aflatoxins was investigated in PBS, certified reference material for AFM1 and in real samples by fluorimetric technique in the microwell plate in presence of various types of competing analytes. Structurally analogous aflatoxins were analyzed using the CR concept. From the assay, it was observed that AFM2 showed the highest CR as compared with AFB1. AFG1 was least recognized by the anti-AFM1 antibodies. The multianalysis of different aflatoxins was also verified by the mixture analysis of AFM1 with AFB1 and AFM2. Assay on microwell plate allowed testing of highly toxic aflatoxins done with low sample volume and with easy handling. Besides the sensitivity and minimal reagent consumption, such multianalysis would lead to simultaneous screening of multiple analytes of aflatoxins.

## Figures and Tables

**Table 1:** Reported biosensing techniques for analysis of AFM1 and AFB1.

Biosensing technique	Analyte	Detection limit	Reference
Surface plasmon resonance (SPR) immunoassay	AFB1	3 ng/mL	[13]
Optical (chemiluminescent) enzyme immunoassay	AFB1	0.01 ng/g	[14]
Long range SPR	AFM1	0.6 pg/mL	[15]
SPR	AFB1	0.2 ng/mL	[8]
Impedance	AFM1	15 ng/L	[16]
Impedance and cyclic voltammetry	AFM1	1 ng/mL	[17]
Bioelectronic recognition assay	AFM1, AFB1	5 pg/mL	[18]
Linear sweep voltammetry	AFM1	0.054 ng/mL	[6]
Direct competitive ELISA (MNPs)	AFM1	8 ng/L	[19]
Indirect competitive ELISA	AFM1	5 ng/L	[20]
Indirect competitive ELISA	AFM1	0.24 ng/mL	[21]
Flow-injection immunoassay	AFM1	11 pg/mL	[22]
Competitive ELISA	AFM1	28 ng/kg	[23]
Indirect competitive ELISA	AFM1	0.04 ng/mL	[24]
Competitive ELISA	AFM1	39 ng/L	[25]
SPR	AFB1, AFG1	0.97 g/kg	[26]
Chemiluminescent ELISA	AFM1	0.05 pg/mL	[27]
Electrochemical impedance spectroscopy	AFM1	1 pg/mL	[28]
Fluorometric ELISA	AFM1, AFM2, AFB1, AFG1	1 pg/mL	This work

**Table 2:** Analytical figures of merit of the fluorometric assay developed for analysis of AFM1.

Analytical parameters	Experimental findings
Linear range	1–50 pg/mL*, 6.25–25 pg/mL^#^
LOD	1 pg/mL
IC_50_	100 pg/mL*, 86 pg/mL^#^
R.S.D.	1.1%*, 0.5%^#^
Adj. *R* ^2^	0.987*, 0.991^#^
Assay sample volume	40 *µ*L
Analysis time	180 min
Sample throughput	128 samples (in triplicate) in 180 min

*PBS; ^#^CRM-BD282.

**Table 3:** Summary of *B*/*B*_0_% of various aflatoxins using standard curve of AFM1.

[AFM1] from standard curve (pg/mL)	*B*/*B*_0_%			
	AFM1	AFM2	AFB1	AFG1
50	61	68	74	94
100	52	62	71	92
150	48	57	N.R.	N.R.
200	45	50	N.R.	N.R.

N.R.: not recorded.

**Table 4:** Recovery studies of real milk sample fortified with different aflatoxins.

Milk samples	Aflatoxin type	Added (pg/mL)	Found (pg/mL)	R.S.D.%	R.E.%	Recovery%
CRM-BD282	AFM1	50	50	0	0	100
CRM-BD282 + S1*	AFM1	50	48.6	47 ± 8	−2.8	97.2
CRM-BD282 + S2*	AFM1	50	46	46.2 ± 1.08	−8	92

CRM-BD282	AFM2	50	45.5	45 ± 0.55	−9	91
CRM-BD282 + S1*	AFM2	50	44.8	44.8 ± 4.44	−10.4	89.6
CRM-BD282 + S2*	AFM2	50	40.2	41 ± 3.75	−19.6	80.4

CRM-BD282	AFB1	50	40.5	40 ± 5	−19	81
CRM-BD282 + S1*	AFB1	50	50.4	50.2 ± 4.96	0.8	100.8
CRM-BD282 + S2*	AFB1	50	46.8	46 ± 4.34	−6.4	93.6

CRM-BD282	AFG1	50	50.2	50 ± 3.2	0.4	100.4
CRM-BD282 + S1*	AFG1	50	46.3	46 ± 1.76	−7.4	92.6
CRM-BD282 + S2*	AFG1	50	46.8	46 ± 4.34	−6.4	93.6

S1*, S2*: real milk samples.
